# Supplementary material for: Input node placement restricting the longest control chain in controllability of complex networks
Source: Sci Rep. 2023 Mar 7;13:3752. doi: 10.1038/s41598-023-30810-w (PMC9992492; doi:10.1038/s41598-023-30810-w)
Supplement: Supplementary file 1 — Supplementary Information. [file 41598_2023_30810_MOESM1_ESM.pdf]

Supplementary Information – Input node placement restricting the longest control chain in controllability of complex networks

Samie Alizadeh,[1] Márton Pósfai,[2] and Abdorasoul Ghasemi[1]

[1]*Department. of Computer Engineering,*
K. N. Toosi University of Technology, Tehran, Iran
[2]*Department of Network and Data Science,*
Central European University, Vienna, Austria

CONTENTS

S1. Computational complexity of the LCC-constrained minimum input problem 2

S2. Efficient integer linear programming formulation of the LCC-constrained minimum input problem 4

S3. Real networks 7

S4. The naïve approximation algorithm 10

References 12

S1. COMPUTATIONAL COMPLEXITY OF THE LCC-CONSTRAINED MINIMUM INPUT PROBLEM

In the following we prove that the computational complexity of the LCC-constrained minimum input problem belongs to the NP-complete class by showing that its corresponding decision problem is both NP and NP-hard[1]. To prove that it is NP, we provide a polynomial-time algorithm to verify for every node set $\mathcal{S}$ whether $\mathcal{S}$ is a valid input node set or not. Proving that it is NP-hard involves reducing the minimum dominating set problem, a known NP-complete problem, to the LCC-constrained minimum input problem, where the reduction is carried out in polynomial time.

Theorem 1. *For a given directed network $\mathcal{G}(\mathcal{V}, \mathcal{E})$ and positive integer $\ell > 0$, a node set $\mathcal{S} \in \mathcal{V}$ is a valid input node set if*

(i) there exists a matching in the bipartite representation $\mathcal{B}$ of network $\mathcal{G}$, such that $\mathcal{S}$ is the set of unmatched nodes;

(ii) and $\mathcal{S}$ is a dominating set in the accessibility graph $\mathcal{G}_\ell$ of network $\mathcal{G}$.

Determining whether a valid input node set $\mathcal{S} \subseteq \mathcal{V}$ exists such that $|\mathcal{S}| = M > 0$ is an NP-complete problem.

Proof. To prove that the problem is NP, we present the following algorithm to check the validity of an input node set $\mathcal{S}$. To check if $\mathcal{S}$ satisfies the matching condition (i), we obtain a pruned bipartite network $\mathcal{B}'$ by taking $\mathcal{B}$ and removing all vertices from $\mathcal{V}^-$ that correspond to vertices in $\mathcal{S}$. If there exists a prefect matching in $\mathcal{B}'$ then there exists a matching in $\mathcal{B}$ such that only nodes in $\mathcal{S}$ are unmatched. We can check whether a perfect matching exists in a bipartite network using, for example, the Hopkroft-Karp algorithm which has worst case runtime $O(\sqrt{|\mathcal{V}|}|\mathcal{E}|)$ [2].

To check the accessibility condition (ii), we fist construct the accessibility graph $\mathcal{G}_\ell$ by calculating the distance between all node pairs in $\mathcal{G}$, which can be done using breath-first search in at most $O\left(|\mathcal{V}|(|\mathcal{V}| + |\mathcal{E}|)\right)$ steps [1]. Verifying that $\mathcal{S}$ is a dominating set in $\mathcal{G}_\ell$ can

be done, for example, by iterating over all edges in $\mathcal{G}_\ell$ which takes at most $O(|\mathcal{V}|^2)$ steps. Consequently, verifying a solution can be done in a polynomial time.

To prove NP-hardness, we reduce the NP-hard minimum dominating set (MDS) problem to our problem [3]. For a given directed network $\mathcal{G}(\mathcal{V}, \mathcal{E})$ a set of nodes $\mathcal{D} \in \mathcal{V}$ is a dominating set if each node in $\mathcal{V}$ is either in $\mathcal{D}$ or has a link pointing at it that starts from a node in $\mathcal{D}$. To reduce the MDS to our problem, we first create an augmented network $\mathcal{G}'$ by adding a self-loop to all nodes in $\mathcal{G}$, solving the LCC-constrained minimum input problem with $\ell = 1$ in $\mathcal{G}'$ is equivalent to solving the MDS in $\mathcal{G}$. To see this, note that the set of self-loops provide a perfect matching in $\mathcal{G}'$; therefore we only have to check that the minimum input set satisfies the accessibility condition. The accessibility graph $\mathcal{G}'_1$ is the same as $\mathcal{G}'$ and self-loops do not affect the dominating sets; therefore the minimum input node set in $\mathcal{G}'$ for $\ell = 1$ is also a minimum dominating set in $\mathcal{G}$.

Therefore, the computational complexity of the LCC-constraint minimum input problem belongs to the NP-complete class. □

S2. EFFICIENT INTEGER LINEAR PROGRAMMING FORMULATION OF THE LCC-CONSTRAINED MINIMUM INPUT PROBLEM

Analyzing the performance of the integer linear programming (ILP) formulation (8) in the main text shows that a na ive implementation of the matching results in poor performance and run-time quickly increases with the number of links in the network (Fig. S2). The performance can be improved by a constant factor using the graph-cycling formulation introduced by Ref. [4].

The basic idea of this approach comes from this idea that a matching partitions the digraph into disjoint paths, see figure (S1). Thus, they partition the digraph into disjoint cycles to find a matching.

We start from a directed graph $\mathcal{G}(\mathcal{V}, \mathcal{E})$ (Fig. S1a). We begin by creating an augmented graph $\mathcal{G}'(\mathcal{V}', \mathcal{E}')$ by adding an auxiliary node x to $\mathcal{G}$, representing the external control signals. We connect this node to all network nodes by a pair of out-going and in-coming links (Fig. S1b). Then, we partition $\mathcal{G}'$ with cycles in a way that cycles are only allowed to overlap at node x (Fig. S1c). Links that participate in cycles in $\mathcal{G}'$ form a matching in $\mathcal{G}$, and the disjoint cycles correspond to disjoint paths in the matching. Therefore the links exiting the auxiliary node as a part of cycle point at input nodes, which are unmatched nodes in the matching (Fig. S1d). Note that there are different ways to partition $\mathcal{G}'$ into disjoint cycles. To find a maximum matching, we aim to partition the $\mathcal{G}'$ with a minimum number of cycles.

To define the associated ILP formulation, we define binary variables $y_{i\to j} \in \{0, 1\}$ corresponding to each link (i, j) in the augmented graph. Here, $y_{i\to j} = 1$ means that (i, j) is a part of cycle partitioning, otherwise, $y_{i\to j} = 0$. To ensure that the solution is a cycle partition, each node v in the network is forced to have exactly one in-coming and one out-going link:

$$\sum_{j\in\mathcal{V}_v{}^+} y_{v\to j} = 1 \tag{S1a}$$

$$\sum_{i\in\mathcal{V}_v{}^-} y_{i\to v} = 1 \tag{S1b}$$

Then, among all possible cycle partitions, we select the one that creates the minimum number of cycles. The number of cycles can be determined by the number of outgoing links from

the auxiliary node that participate in cycles. Thus, we minimize the $\sum_{j\in\mathcal{V}} y_{x\to j}$ objective function. Note that the number of out-going and in-coming links of the auxiliary node must be equal, thus they have to satisfy the $\sum_{j\in\mathcal{V}} y_{x\to j} = \sum_{i\in\mathcal{V}} y_{i\to x}$ constraint. Next, we should ensure the accessibility. Thus, for each node i, the auxiliary node must connect to at least one node of set $\mathcal{V}_i^\ell$, where $\mathcal{V}_i^\ell$ is the set of nodes from where we can reach node i in at most ℓ steps. This is enforced by the $\sum_{j\in\mathcal{V}_i^\ell} y_{x\to j} \geq 1$ constraint. Putting it all together, we obtain the following ILP formulation:

$$\min_{y_{i\to j}\in\mathcal{E}'} \quad \sum_{j\in\mathcal{V}} y_{x\to j} \tag{S2a}$$

subject to

$$y_{i\to j} \in \{0,1\} \qquad \forall\,(i\to j)\in\mathcal{E}' \tag{S2b}$$

$$\sum_{j\in{\mathcal{V}_v}^+} y_{v\to j} = 1 \qquad \forall\, v = 1, ..., N \tag{S2c}$$

$$\sum_{i\in{\mathcal{V}_v}^-} y_{i\to v} = 1 \qquad \forall\, v = 1, ..., N \tag{S2d}$$

$$\sum_{j\in\mathcal{V}_v^\ell} y_{x\to j} \geq 1 \qquad \forall\, v = 1, ..., N \tag{S2e}$$

$$\sum_{j\in\mathcal{V}} y_{x\to j} = \sum_{i\in\mathcal{V}} y_{i\to x} \tag{S2f}$$

Figure S2 compares the performance of the na ive ILP formulation (8) and the graph-cycling implementation in Eq. (S2). We measure the run-time by the number of nodes searched in the branch-and-bound tree to solve the problem. We find that although the run-time for both formulations grows exponentially, the graph-cycling implementation is significantly faster, allowing us to explore larger network instances.

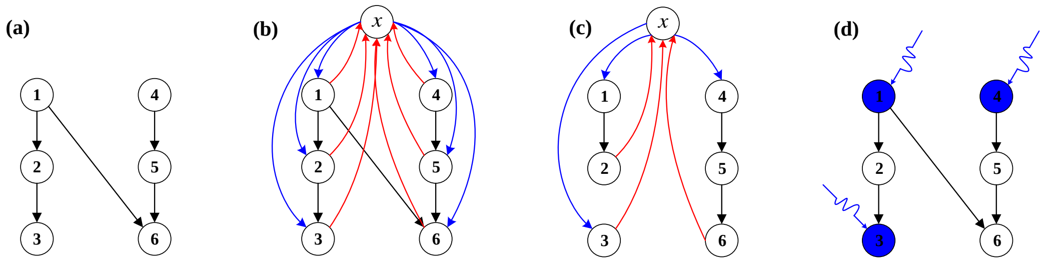

FIG. S1. **Graph-cycling ILP formulation. (a)** A small directed network. **(b)** We create an augmented graph by adding an auxiliary node and connecting it to all nodes via a pair of in-coming and out-going links. **(c)** The partitioned graph for $\ell = 1$. Here, the directed graph is partitioned into three disjoint cycles. **(d)** Input nodes are the nodes that have a link pointing at them that originates from the auxiliary node and is part of a cycle.

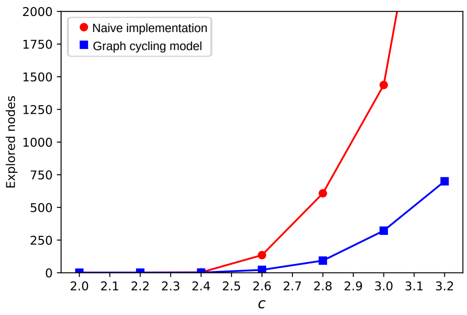

FIG. S2. **Comparing the performance of the ILP implementations**. We measure the run-time of the models by the number of nodes searched by the ILP solver in the branch-and-bound tree. We explored ER networks with $N = 500$ nodes. Each data point represents an average of 100 independent instances.

S3. REAL NETWORKS

The descriptions of the datasets and the results of created core and required cost for different LCC constraints are summarized in Table SI and SII.

TABLE SI. The collection of real networks. We provide the number of nodes N, number of links L, average degree c, degree heterogeneity $H = \max(H^{in}, H^{out})$, where $H^{in/out}$ can be defined by $H^{in/out} = \frac{1}{cN^2}\sum_i \sum_j (k_i^{in/out} - k_j^{in/out})$[5], c is a constant, and $k_i^{in/out}$ shows input/output degree of node.

Networks		N	L	c	H	Ref.
Electric circuits	s208	122	189	1.5	.63	[6]
	s838	512	819	1.5	.64	[6]
Food Web	Baywet	128	2,106	16.4	.8	[7]
	Mangwet	97	1,492	15.3	.98	[8]
	Ythan	135	601	4.4	1.42	[9]
	Littlerock	183	2,494	13.6	1.36	[9]
Transcript	E.Coli	418	519	1.2	1.8	[6]
	Yeast	688	1,079	1.5	1.84	[6]
Web of Trust	CentralCoast	943	1,227	1.3	1.7	[10]
	Napa-rev	646	926	1.4	1.7	[10]
Metabolic	C.Elegans	1,173	2,864	2.4	.7	[11]
	Yeast	1,511	3,833	2.5	.78	[11]
Airport	USairport	1,574	28,236	17.9	1.51	[12]
Web	polblogs-rev	1,224	19,022	15.5	1.51	[11]
	Stanford	281,903	2,312,497	8.2	1.26	[13]
Social	prisoninmate	67	182	2.7	.81	[11]
	Epinions1	75,879	508,524	6.7	1.71	[14]
	WikiVote	7,115	139,311	14.5	1.69	[15]
Communication	Email	265,214	420,045	1.5	1.58	[16]
	WikiTalk	2,394,385	5,021,410	2.1	.14	[15]
Citation	HepPh	34,546	420,045	12.2	1.58	[17]
	HepTh	27,770	352,807	12.7	1.7	[17]
Peer to Peer	Gnutella31	62,586	147,892	2.3	.88	[18]

TABLE SII. The result of created core and required cost in real networks for different LCC constraints.

	$\ell = 1$			$\ell = 2$			$\ell = 3$		
Networks	$n_{\rm i}(\ell)$	$n_{\rm core}$	$C(\ell)$	$n_{\rm i}(\ell)$	$n_{\rm core}$	$C(\ell)$	$n_{\rm i}(\ell)$	$n_{\rm core}$	$C(\ell)$
s208	.46	.45	.22	.31	.38	.08	.29	.30	.04
s838	.45	.48	.22	.33	.46	.09	.29	.42	.06
Baywet	.28	.91	.03	.28	.67	.007	.26	.38	.007
Mangwet	.30	.84	.07	.31	.55	.01	.31	.31	0
Ythan	.52	.24	.01	.51	.04	0	.51	.04	0
Littlerock	.66	.85	.01	.65	.85	.01	.65	.85	.005
E.Coli	.74	0	0	.74	0	0	.74	0	0
Yeast	.82	.001	0	.82	.001	0	.71	.001	0
CentralCoast	.77	.003	.006	.76	0	0	.76	0	0
Napa-rev	.77	0	.001	.77	0	0	.77	0	0
C.Elegans	.28	.65	.14	.33	.64	.03	.30	.50	.03
USairport	.44	.76	.07	.38	.13	.01	.37	.09	.001
polblogs	.37	.37	.01	.35	.006	0	.35	.005	0
Stanford	.45	.81	.14	.37	.72	.05	.35	.61	.05
prisoninmate	.34	.76	.19	.19	.74	0.04	.16	.71	.02
Epinions1	.63	.12	.08	.56	.04	.01	.55	.02	0
WikiVote	.67	.0007	.007	.70	0	0.0001	.70	0	0
Email	.92	.01	.001	.92	0.00001	.0008	.92	0	0
WikiTalk	.96	.001	.0001	.96	0	0	.96	0	0
HepPh	.30	.23	.07	.24	.12	.008	.23	.09	.003
HepTh	.29	.40	.07	.23	.23	.01	.22	.20	.008
Gnutella31	.73	.60	.0001	.73	.0005	0	.73	.0005	0

S4. THE NAÏVE APPROXIMATION ALGORITHM

Here we introduce a naïve greedy algorithm to approximate the LCC-constrained minimum input problem, and we compare its performance to the approximation algorithm introduced in the main text Sec. 4. In the main text we showed that the input nodes that ensure LCC-constrained structural controllability have to satisfy both the matching and the accessibility constraints (Sec. 3), i.e., (i) there exists a matching such that only nodes representing inputs are unmatched in $\mathcal{B}$ and (ii) all nodes are at most ℓ steps from input nodes in $\mathcal{G}_\ell$. Therefore, a naïve approach to find a valid input node set is to identify a maximum matching in $\mathcal{B}$ and a minimum dominating set in $\mathcal{G}_\ell$ independently using the greedy algorithms described in Sec. 4.1 and 4.2, respectively. A valid input node set is then provided by the union of the unmatched nodes in $\mathcal{B}$ and the dominating nodes in $\mathcal{G}_\ell$. We compare the performance of the naïve greedy algorithm to the exact solution in Fig. S4, finding that the naïve algorithm performs poorly in most cases except for networks that require a very large number of inputs. In the worst case, the estimated number of input nodes is 3.6 times larger than the optimal, while the approximation algorithm in the main text estimates $N_{\mathrm{i}}(\ell)$ within 5% error for the same analyzed networks.

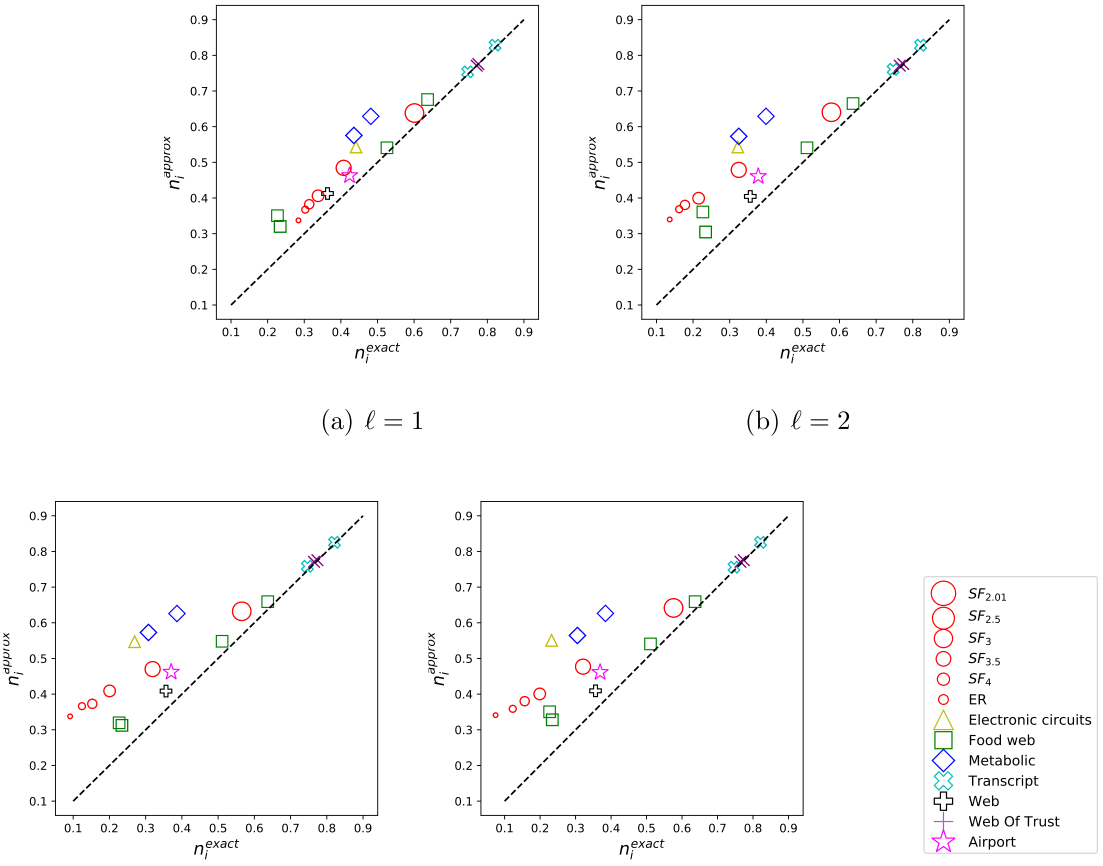

(a) $\ell = 1$ (b) $\ell = 2$

(c) $\ell = 3$ (d) $\ell = 4$ (e)

FIG. S3. **The performance of the naive approximation algorithm to determine $N_{\mathbf{i}}(\ell)$.** We compare the approximate fraction of input nodes to the exact solution for model and real networks with a few hundred nodes. The synthetic networks are SF and ER model networks with $N = 1000$ and average degree $c = 4$. Each data point is an average of 100 independent networks.

[1] T. H. Cormen, C. E. Leiserson, R. L. Rivest, and C. Stein, *Introduction to algorithms* (MIT press, 2009).

[2] J. E. Hopcroft and R. M. Karp, An nˆ5/2 algorithm for maximum matchings in bipartite graphs, SIAM Journal on computing **2**, 225 (1973).

[3] J. Hartmanis, Computers and intractability: a guide to the theory of np-completeness (michael r. garey and david s. johnson), Siam Review **24**, 90 (1982).

[4] F. L. Iudice, F. Garofalo, and F. Sorrentino, Structural permeability of complex networks to control signals, Nature communications **6**, 1 (2015).

[5] Y.-Y. Liu, J.-J. Slotine, and A.-L. Barabási, Controllability of complex networks, nature **473**, 167 (2011).

[6] R. Milo, S. Shen-Orr, S. Itzkovitz, N. Kashtan, D. Chklovskii, and U. Alon, Network motifs: simple building blocks of complex networks, Science **298**, 824 (2002).

[7] D. Baird, J. Luczkovich, and R. R. Christian, Assessment of spatial and temporal variability in ecosystem attributes of the st marks national wildlife refuge, apalachee bay, florida, Estuarine, Coastal and Shelf Science **47**, 329 (1998).

[8] N. D. Martinez, Artifacts or attributes? effects of resolution on the little rock lake food web, Ecological monographs **61**, 367 (1991).

[9] R. E. Ulanowicz and D. L. DeAngelis, Network analysis of trophic dynamics in south florida ecosystems, US Geological Survey Program on the South Florida Ecosystem **114**, 45 (2005).

[10] M. A. Levy and M. N. Lubell, Innovation, cooperation, and the structure of three regional sustainable agriculture networks in california, Regional environmental change **18**, 1235 (2018).

[11] H. Jeong, B. Tombor, R. Albert, Z. N. Oltvai, and A.-L. Barabási, The large-scale organization of metabolic networks, Nature **407**, 651 (2000).

[12] T. Opsahl, F. Agneessens, and J. Skvoretz, Node centrality in weighted networks: Generalizing degree and shortest paths, Social networks **32**, 245 (2010).

[13] J. Leskovec, K. J. Lang, A. Dasgupta, and M. W. Mahoney, Community structure in large networks: Natural cluster sizes and the absence of large well-defined clusters, Internet Mathe-

matics **6**, 29 (2009).

[14] M. Richardson, R. Agrawal, and P. Domingos, Trust management for the semantic web, in *International semantic Web conference* (Springer, 2003) pp. 351–368.

[15] J. Leskovec, D. Huttenlocher, and J. Kleinberg, Signed networks in social media, in *Proceedings of the SIGCHI conference on human factors in computing systems* (2010) pp. 1361–1370.

[16] J. Leskovec, J. Kleinberg, and C. Faloutsos, Graph evolution: Densification and shrinking diameters, ACM transactions on Knowledge Discovery from Data (TKDD) **1**, 2 (2007).

[17] J. Leskovec, J. Kleinberg, and C. Faloutsos, Graphs over time: densification laws, shrinking diameters and possible explanations, in *Proceedings of the eleventh ACM SIGKDD international conference on Knowledge discovery in data mining* (2005) pp. 177–187.

[18] M. Ripeanu, I. Foster, and A. Iamnitchi, Mapping the gnutella network: Properties of large-scale peer-to-peer systems and implications for system design, arXiv preprint cs/0209028 (2002).
